# Effective Strategies for Monitoring and Regulating Chemical Mixtures and Contaminants Sharing Pathways of Toxicity

**DOI:** 10.3390/ijerph120910549

**Published:** 2015-08-28

**Authors:** Arjun K. Venkatesan, Rolf U. Halden

**Affiliations:** Center for Environmental Security, The Biodesign Institute, Global Security Initiative, Arizona State University, Tempe, AZ 85287, USA; E-Mail: avenka21@asu.edu

**Keywords:** environmental monitoring, flame retardants, regulatory framework, chemical mixtures, toxicity

## Abstract

Traditionally, hazardous chemicals have been regulated in the U.S. on a one-by-one basis, an approach that is slow, expensive and can be inefficient, as illustrated by a decades-long succession of replacing one type of organohalogen flame retardants (OHFRs) with another one, without addressing the root cause of toxicity and associated public health threats posed. The present article expounds on the need for efficient monitoring strategies and pragmatic steps in reducing environmental pollution and adverse human health impacts. A promising approach is to combine specific bioassays with state-of-the-art chemical screening to identify chemicals and chemical mixtures sharing specific modes of action (MOAs) and pathways of toxicity (PoTs). This approach could be used to identify and regulate hazardous chemicals as classes or compound families, featuring similar biological end-points, such as endocrine disruption and mutagenicity. Opportunities and potential obstacles of implementing this approach are discussed.

## 1. Introduction

Today, more than 84,000 chemicals are included in the U.S. Toxic Substances Control Act (TSCA) inventory, and approximately 10% of these chemicals are associated with cancer [1,2]. The majority of current environmental and biological monitoring strategies rely on chemical analysis of individual target compounds. Similarly, today’s chemical regulations in the U.S. and many other countries worldwide target specific chemicals rather than groups of compounds posing threats via specific pathways of toxicity that result in shared adverse outcomes in human health. Contrary to the single-chemical scenarios that inform risk assessments today, people in everyday life get exposed to multiple chemicals, which may exert cumulative effects on humans or interact in unpredictable ways via chemical interactions [1]. One major concern with chemical mixtures is the possibility of inducing synergistic effects, whose overall effects exceed the sum of adverse impacts caused by individual exposures. Synergistic effects resulting from the inhalation of radon progeny and smoking, and exposure to asbestos and smoking have been documented for lung cancer incidents [3,4]. Two classic approaches to address toxicity resulting from chemical mixtures are concentration addition (CA) and independent action (IA), for which information on both the individual chemical toxicity and concentration of the chemical in the respective mixture of interest is needed [5,6]. For example, the standard method for assessing mixture toxicity and exposures of dioxin-like compounds makes use of Toxic Equivalency Factors (TEF). This methodology expresses the composite chemical risk resultant from complex mixtures of dioxin-like compounds (polychlorinated/polybrominated dioxins, furans and biphenyls), in a single value, using as a benchmark the most toxic form of dioxin, *i.e.*, 2,3,7,8-tetrachlorodibezo-*p*-dioxin (TCDD). The approach assumes that all dioxin-like compounds exhibit a similar profile of effect on the aryl hydrocarbon receptor (AhR), and that the effects are additive. However, research has shown that the presence of certain congeners (e.g., PCB 153) in mixtures antagonized the effects of TCDD, highlighting some major limitations for the use of additive TEF approach [7]. In addition, these approaches of estimating toxicity require extensive ecotoxicological data for individual chemicals and a thorough characterization of the chemical mixtures. An added concern is the presence of unknown production impurities in chemicals and unrecognized transformation products for which no current methods of detection are available, with these toxic analogs contributing to the observed toxicity. 

One important class of chemicals that exhibit mixture toxicity in the environment is the group of organohalogen flame retardants (OHFRs), particularly the various congeners of polybrominated diphenyl ethers (PBDEs). Among these, the large number of congeners, their varying toxicity (depending on the degree of halogenation), the possibility of inter-transformation of congeners from a higher to a lower halogenation state, and presence of impurities (such as dioxins and furans), make monitoring and legislative actions for OHFRs exceptionally complicated. Managing this group of problematic chemicals has proven to be a challenge, as indicated by 45 years of still ongoing legislative actions for PBDEs. Relevant challenges and legislative initiatives are highlighted in the following, using OHFRs as an illustrative case study of lessons learned from monitoring practices and chemical regulations.

## 2. Organohalogen Flame Retardants and Current Regulations 

**Figure 1:**
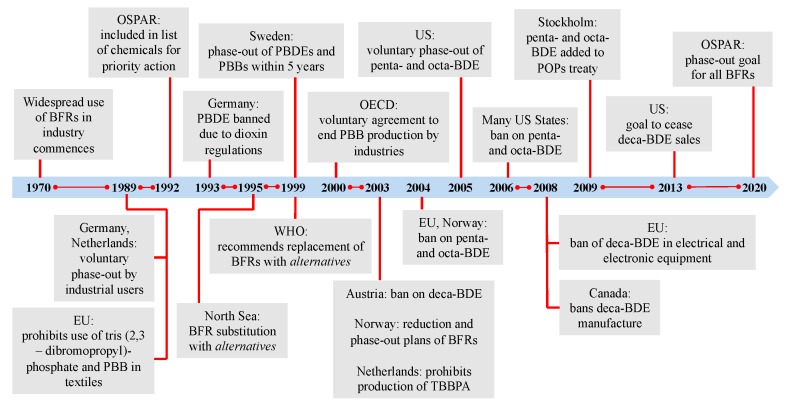
Timeline of legislative initiatives for brominated flame retardants spanning half a century of widespread use in industries. EU: European Union; OECD: Organisation for Economic Co-operation and Development; OSPAR: Oslo and Paris Conventions for the Protection of the Marine Environment of the North-East Atlantic; PBB: polybrominated biphenyls; PBDE: polybrominated diphenylether; POP: persistent organic pollutant; TBBPA: tetrabromobisphenol A; WHO: World Health Organization.

Many OHFRs are known to be persistent, bioaccumulative and toxic, while also having been detected globally in the environment, in wildlife, and in human populations [8]. Following these concerns, two major commercial formulations of flame retardants, namely penta- and octa-brominated diphenyl ethers (BDEs), were banned by the European Union (EU) in 2002; the fully brominated deca-BDE later also was banned from use in electrical and electronic applications within the EU in 2008 [9,10]. In the U.S., penta- and octa-BDEs were voluntarily phased-out in 2005 and deca-BDE sales were expected to cease by the end of 2013, with definitive numbers still pending. Regulating brominated flame retardants is and has been an ongoing challenge for the past 45 years, ever since their widespread use began in the 1970s (Figure 1). Even after ban and phase-out initiatives, many products manufactured with penta- through deca-BDEs prior to regulatory actions remain in use today. Replacement chemicals now finding their way into commerce are structurally similar to the original, now banned or phased-out compounds; not surprisingly, they show similar characteristics, including persistence, bioaccumulation potential and toxicity in the environment [9,11]. In general, all these chemicals feature one or more benzene rings with varying degrees of halogenation, a basic chemical structure endowing banned polychlorinated biphenyls (PCBs) and carcinogenic polychlorinated dibenzo-*p*-dioxins with their marked toxicity and endocrine disrupting characteristics. Four decades after being banned in the U.S., PCBs still are detected in most environments, wildlife and human populations, and BDE formulations are on a trajectory to persist for many decades following their recent ban [12,13]. In addition, the realization has set in more recently, that these mass produced OHFRs are transformed in the environment abiotically and biotically to various toxic transformation products, such as mono- and polyhydroxylated congeners of the manufactured parent compound [14,15,16]. Furthermore, structurally related impurities of similar toxicity have been known for quite some time to be present in commercial grade flame retardants used in the manufacturing of consumer products [17].

The solution to the problem of persistent chemical pollution ultimately is to curtail the use and production of compounds sharing a structural and functional similarity to known hazardous compounds. The futility of making minor modifications to the carbon backbone or halogen substitution pattern of persistent OHFRs and then hoping for a different, better outcome has been demonstrated over and over again. Pragmatic and prudent steps for reducing the unwanted environmental pollution and adverse human health impacts these chemicals pose include:

- Avoiding the use of flame retardants in consumer products that do not pose a significant fire hazard in the first place;
- Avoiding the use of flame retardants that resemble in molecular size and structure their harmful predecessors;
- Developing for use in products that absolutely require them, next-generation flame retardants featuring the following characteristics while still delivering the desired flame retardation qualities:
  ○ covalently bound to the consumer products to minimize release and exposure, wherever possible and practical;
  ○ large molecular size (e.g., polymers) that limits and ideally precludes uptake by biota following environmental release [18];
  ○ absence of size and shape characteristics contributing to affinity and binding to human and animal receptors, thereby reducing the risk of toxicity, for example, of endocrine disruption via binding to hormone receptors.

A recent study by our group featured the use of municipal sewage sludge (a solid byproduct of wastewater treatment) for identifying and prioritizing persistent and bioaccumulative chemicals. Eight top priority chemicals of high abundance and bioaccumulative potential were identified; these included five organohalogens of which three were brominated flame retardants (BFRs) [19]. The BFRs were the third most abundant group of chemicals detected in nationally representative U.S. sewage sludges (among 239 compounds surveyed), suggesting their widespread use and ongoing exposures of human populations and wildlife. Also, the study revealed that among 55 potentially bioaccumulative chemicals detectable in sewage sludge, 93% were halogenated. It is well known that the replacement of hydrogen with halogen atoms is positively correlated with an increase in the chemical’s hydrophobicity, persistence and toxicity [20]. Our analysis of compounds included in the U.S. Safe Drinking Water Act of the U.S. EPA showed that 74 percent of regulated organic compounds carry at least one and more frequently multiple halogen substituents.

With these facts firmly established, the following question arises: why are we continuing to design, manufacture and use organohalogen compounds that are known to be hazardous as a class or family? Instead of regulating individual formulations like penta- or octa-BDEs and replacing those with similar formulations like deca-BDE, a more proactive and protective strategy is required in chemical safety management. There is a need to recognize and circumvent the root cause that endows organic compounds, such as OHFRs, with the widely manifested potential for adverse effects, specifically persistence, bioaccumulation potential and toxicity.

## 3. Future Monitoring Strategies

Future monitoring approaches can pave the way to creating and enforcing better regulations. They should include both chemical analyses and specific bioassays to identify anthropogenic chemical agents eliciting unwanted and unnecessary toxicity in the environment. These agents then could be targeted by more effective regulations zeroing in on chemical groups featuring similar modes of action (MOAs) and pathways of toxicity (PoTs). Techniques that identify specific interactions of chemical mixtures with human receptors or biological end-points (e.g., binding to thyroid hormone receptor and resultant endocrine disruption) will allow for an identification of biologically active groups of chemical in the environment. Examples of such approaches include effects-directed analysis (EDA), toxicity identification evaluation (TIE) and other pathway-based cellular and molecular assays [21,22,23]. EDA uses bioassays (typically *in vitro*) as tools to narrow down and sensitively detect organic chemical mixtures in the environment that contribute to an observed and measureable toxic effect. This is achieved by fractionation based on physical-chemical properties of the components to reduce the complexity of contaminant mixtures in the sample into prioritized sub-groups. TIE relies on whole-organism (e.g., daphnids, mysids) toxicity testing to enable a characterization of chemical mixtures into sub-classes, followed by identification and validation. EDA has the advantage of having high specificity in toxicant identification, since specific biological endpoints are studied via *in vitro* testing. Whereas TIE lacks specificity due to whole organism testing, it does however consider toxicant bioavailability considerations in the analysis. Currently, the U.S. EPA requires chemical testing from manufacturers and processors only if: (*i*) a chemical may present an unreasonable risk of injury to human health or environment; (*ii*) the chemical is produced in a substantial quantity that could result in significant environmental releases and human exposures; and/or (*iii*) the available data is inadequate [24]. However, these criteria may exclude many chemicals of low production volume, other transformation products, unidentified chemicals and chemical mixtures that might be of significant concern to human health and the environment. Hence, incorporating EDA/TIE like bioassays into monitoring programs would be a prudent and promising approach to identify risks of injury to human health or the environment from chemical mixtures including unknowns, whose occurrence and harmful potential cannot be established purely on the basis of chemical monitoring of singular compounds.

The U.S. EPA’s Computational Toxicology (CompTox) research aims to integrate advances in molecular biology, toxicology, and computer science to overcome limitations of traditional laboratory animal-based toxicity tests and to help prioritize chemicals through high-throughput screening assays via Toxicity Forecaster (ToxCast). This program relies on cell-based bioassays for screening of chemicals and to develop predictive models for health outcomes [25]. Application of such cell-based bioassays for water quality assessment has been successfully carried out in the past [26,27,28]. One study tested six endpoints of particular importance to human and environmental health (genotoxicity, estrogenicity, neurotoxicity, phytotoxicity, dioxin-like activity, and non-specific cell toxicity) to establish the efficacy of different water treatment barriers [26]. The authors applied the concept of TEQ by selecting one reference compound in each of the tests. The study concluded the bioanalytical tools to be reproducible and robust, and found the TEQ approach to be useful for assessing the removal of micro-pollutants in various treatment steps. Another recent study examined the suitability of cell-based bioassays to benchmark water quality and tested 103 unique *in vitro* bioassays, including induction of xenobiotic metabolism, specific and reactive modes of toxic action, as well as activation of adaptive stress response pathways and system responses [28]. The study concluded that an ideal battery of bioassays should include sensitive bioassays covering a wide range of cellular toxicity pathways for effective water quality assessment. The widely applied AhR activation endpoint was found to be a relevant indicator of presence of chemicals and was suggested to be included in any routine battery of tests. Specific receptor-mediated modes of action—including effects related to estrogenic, glucocorticoid and antiandrogenic pathways—showed the most promise for water quality screening applications. Additionally, the study identified oxidative stress response as a highly sensitive and selective indicator of environmental pollution [28]. 

Once a toxic hazard has been established from use of the above screening tools, the key to successful characterization of chemical or chemical mixtures are analytical tools such as liquid/gas chromatography-mass spectrometry (LC/GC-MS) as well as computer-based tools for chemical structure elucidation. Non-target analysis by GC-MS in scan mode followed by mass spectral analysis using database searches can aid in identifying the unknowns present in the mixture that may significantly contribute to the observed toxicity. Structural elucidation and chemical identification can be aided greatly by using high resolution mass spectrometry (HRMS) [29]. In addition to instrumental techniques, programs such as quantitative structure-activity relationship (QSAR) have also been used in the past to predict and estimate the toxicity of chemical mixtures [30]. Although the latter tool can aid in identifying similar MOA of chemicals, the output from QSAR depends on proper input parameters and fails to address the problem of mixtures. Whereas this restricts the quantitative application of QSARs in environmental monitoring, the approach is quite useful for prioritizing and identifying chemicals that share common MOA. Combining EDA-like strategies with *in silico* analytical tools can further complement the traditional analysis of target compounds in environmental matrices. Combining these approaches then enables an identification and quantitation of harmful effect exerted by both single chemicals (including manufacturing byproducts and chemical degradates of commercial formulations) and chemical mixtures (like BFRs and other structurally similar organohalogens) featuring similar modes of toxic action. Such information then could inform both the design and regulation of industrial chemicals as well as enforcement of regulations leveled for environmental and human health protection [31]. However, bioassays are expensive and cannot replace the traditional monitoring approach when used in isolation. These tests should be included to identify chemicals causing an observed toxicity in environmental samples or where there is a high probability of toxicity to environmental end points, e.g., soils amended with contaminated sewage sludge and surface water receiving wastewater inputs. Though incorporating such techniques for regular monitoring is difficult and not economically feasible, testing samples yearly or biennially will benefit the regulatory community in prioritizing contaminants for future monitoring needs and to evaluate the effectiveness of legislative actions.

## 4. Conclusions 

Polybrominated mono and bi-nuclear aromatic flame retardants of low molecular weight are prone to pose significant environmental and human health risks, no matter what minor modifications are made to the structure of their carbon backbone and its halogen substitution pattern. Learning from past failures and improving on existing monitoring strategies is a necessity for creating and implementing an effective regulatory framework and, ultimately, for designing more sustainable chemicals. Discontinuing unnecessary uses of brominated flame retardants and developing non-halogenated alternatives may serve as the starting point for arriving at safer, greener and more sustainable consumer products. Fire retardation has essential applications in society but does not need to come at the expense of contaminating and harming the environment and human populations for multiple generations into the future. Analytical chemists and toxicologists have an opportunity to aid in the transition toward a more sustainable chemical future, by devising assays that capture multiple bad chemical players and that provide a composite measure for the toxicity these exert. Inclusion of such assays in routine monitoring and regulatory framework may provide opportunities to avoid harmful chemicals that are similar to legacy toxicants and to shorten the time taken for legislative actions, which on average takes some 14 years following first reports of significant risks posed [32]. 
